# Toxicity determinants of multi-walled carbon nanotubes: The relationship between functionalization and agglomeration

**DOI:** 10.1016/j.toxrep.2016.01.011

**Published:** 2016-01-19

**Authors:** Manfredi Allegri, Dimitrios K. Perivoliotis, Massimiliano G. Bianchi, Martina Chiu, Alessandra Pagliaro, Malamatenia A. Koklioti, Aikaterini-Flora A. Trompeta, Enrico Bergamaschi, Ovidio Bussolati, Constantinos A. Charitidis

**Affiliations:** a Unit of General Pathology, Department of Biomedical, Biotechnological and Translational Sciences, University of Parma, 43125 Parma, Italy; b Research Unit of Advanced, Composite, Nano-Materials and Nanotechnology, Department of Materials Science and Engineering, School of Chemical Engineering, National Technical University of Athens, GR-157 80 Athens, Greece; c Unit of Occupational and Environmental Medicine, Department of Clinical and Experimental Medicine, University of Parma, 4312 Parma, Italy

**Keywords:** BET, Brunauer, Emmett and Teller, BSA, Bovine Serum Albumin, CFE, colony forming efficiency, CNT, carbon nanotubes, CVD, carbon vapor deposition, DMEM, Dulbecco’s modified Eagle’s medium, DTT, dithiothreitol, EDS, energy dispersive X-ray spectrometry, FBS, Fetal Bovine Serum, FT-IR, Fourier transform infrared spectroscopy, MWCNT, multi-walled carbon nanotubes, NO, nitric oxide, SDS, sodium dodecyl sulphate, SDS-PAGE, SDS polyacrylamide gel electrophoresis, SWCNT, single-walled carbon nanotubes, SSA, specific surface area, TEER, Trans-Epithelial Electrical Resistance, TGA, thermogravimetric analysis, XRD, X-ray diffraction, Carbon nanotubes, Macrophages, Airway epithelium, Inflammation, Functionalization, Agglomeration, Protein corona

## Abstract

The elucidation of toxicity determinants of multi-walled carbon nanotubes (MWCNT) is still incomplete. Functionalization with carboxyl groups is, however, commonly used to mitigate MWCNT toxicity, although the rationale for the mitigating effect has not been fully clarified yet. In this work, two optimized chemical vapor deposition methods were employed to obtain MWCNT of comparable length but different diameter, which were subsequently functionalized. For MWCNT of diameter larger than 40 nm, no detrimental effects on cell viability of macrophages were observed, while mild cytotoxicity was recorded for diameters between 15 and 40 nm, with a mitigating effect of functionalization. To investigate the factors responsible for the mitigation, we used the thinnest MWCNT preparation on different cell models, evaluating several endpoints, such as viability, production of nitric oxide (NO), expression of pro-inflammatory markers, the Trans-Epithelial Electrical Resistance (TEER), and clonogenic activity. Substantial mitigation of the changes caused by pristine MWCNT was observed not only with carboxyl- but also with amino-functionalized MWCNT, suggesting that negative or positive surface charge was not the main factor responsible for the effect. Instead, either functionalized preparation exhibited a stronger tendency to agglomerate that was strictly dependent on the presence of proteins. Moreover, we found that either carboxyl- or amino-functionalized MWCNT adsorbed a larger amount of serum proteins than pristine counterparts, with a distinctive pattern for each type of MWCNT. We propose, therefore, that the formation of larger agglomerates, dependent upon different protein coronae, contributes to mitigate the biological effects of functionalized MWCNT in protein-rich biological media.

## Graphical abstract

**Figure:**
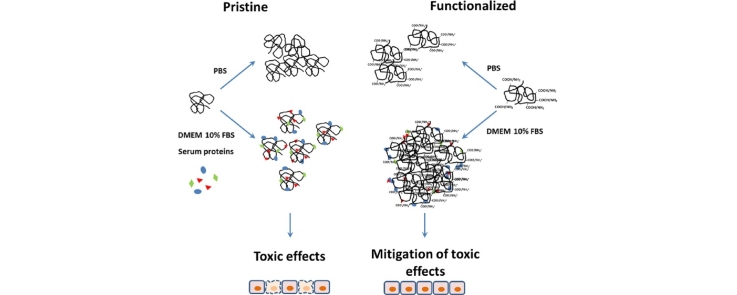

## 1 Introduction

After their discovery by Iijima [29], carbon nanotubes (CNT) have been increasingly used in advanced industrial applications. Indeed, due to their excellent physico-chemical, electrical and mechanical properties, they are applied in numerous technological fields such as polymer composites, microelectronics, energy storage and sensors [18]. CNTs are graphitic hollow filaments of variable lengths, up to several hundred micrometers, depending on the production method. Single-walled carbon nanotubes (SWCNT) are composed of a single cylindrical sheet of graphene, while multi-walled carbon nanotubes (MWCNT) consist of several concentric, coaxial, rolled up graphene sheets [44]. The CNT diameter typically ranges from 0.4 to 3 nm for SWCNT and from 1.4 to 100 nm for MWCNT [62]. Chemical vapor deposition (CVD) is the most frequently used method for CNT synthesis and is the prevalent technique for mass production of CNT due to its easy scaling-up [9].

Different preparations of MWCNT yield contrasting results about their bio-safety, as some preparations appear to be highly hazardous, while others seem harmless [13], [24], [47], [51]. Thus, toxicity evaluation of these materials has to be taken on a case-by-case study, because CNT cannot be regarded as a simple chemical substance. Therefore, the investigation of MWCNT toxicity has to be designed according to their specific features and cannot adopt the same strategy of the conventional toxicology studies applied for general chemical compounds [42].

The conventional view point is that SWCNT exhibit significant cytotoxicity to human and animal cells through various mechanisms [12], [33], [40], whereas MWCNT are considered less active [32]. However, increasing evidences suggest that MWCNT can be inflammogenic and fibrogenic in rodents [11], [20]. Moreover, a specific MWCNT preparation (MWCNT-7) has been recently classified as possibly carcinogenic to humans (Group 2B) by the International Agency for Research on Cancer, although other preparations of MWCNT were not classifiable respect to their carcinogenicity to humans [26]. These contrasting results can be attributed to the use of materials with different degree of purity and structural features [68], as well as to the conditions adopted for the *in vitro* studies [72] and the cell types tested for the assays [35]. Thus, despite several excellent reviews on the advancement in knowledge about CNT toxicity [2], [16], [19], [36], [37], [46], [63], the current understanding of the physico-chemical determinants of MWCNT toxicity is still incompletely settled [30].

In general, the toxicity of CNTs is attributed to their physico-chemical characteristics, such as length, diameter, shape, purity, surface area and surface chemistry [37], [38], which, in turn, are remarkably influenced by the synthetic route. CNT contamination by catalyst residues is unavoidable during their production, so that the impact of residual catalyst metals may be also important [38]. Among the various determinants known to influence the biological activity of CNT, also functionalization with carboxylic groups has been considered and investigated. However, contrasting results have been reported in this case too. Functionalization enhances toxicity in airway epithelial BEAS-2B cells [10], [65], while on the contrary, it has been demonstrated to suppress bioactivity in other epithelial models [39] and in macrophages [28]. Also *in vivo* models have yielded diverging data, with mitigating [31], [58] or enhancing [64] effects reported for different endpoints.

When suspended in biological media, MWCNT adsorb macromolecules that form a corona and modify the properties of the material [41]. Protein adsorption is heavily influenced by surface curvature and surface area. Surface curvature is directly related to the outer diameter [27], [52], while surface area is influenced by outer diameter, pore volume and surface chemistry. In particular, surface chemistry influences the adsorption of macromolecules to CNT depending on: (1) the available specific surface area (functionalized CNT may present higher or lower surface area values according to the functional groups which are attached on their sidewalls); (2) the solution pH, in relation to CNT pKa value (at pH below p*K*_α_ adsorption is increased); (3) the ionic strength in the solution (ionic strength increases adsorption) [14]. However, the relationship between protein corona, functionalization and MWCNT biological effects is still under investigation.

In this work, we tested the hypothesis that functionalization with carboxyl or amino groups affects the formation of protein corona and, hence, the biological effects of MWCNT. To this purpose, we preliminary examined four MWCNT preparations, three synthesized in our laboratory *via* CVD method and one commercial, of comparable length but different diameter and surface chemistry, and compared their effects on cell viability with those exhibited by a benchmark MWCNT preparation obtained from the Joint Research Centre (JRC) repository (Ispra, Varese, Italy). One of the preparations exhibiting changes in biological activity attributable to functionalization was then further investigated, studying both protein corona and several toxicological endpoints.

## 2 Experimental procedures

### 2.1 Supply and synthesis of carbon nanotubes

A thermal chemical vapor deposition (CVD) reactor was used to synthesize MWCNΤs. The reactor consists of a horizontal quartz tube with 3.4 cm inner diameter and 100 cm length housed in a three-zone 80 cm long cylindrical furnace. Synthesis of CNT was performed by two approaches. In the first case, camphor and ferrocene were used as carbon source and catalyst, respectively, while acetylene as carbon source and iron particles supported on Al_2_O_3_ substrate as catalyst were used in the latter.

More specifically, for the preparation of the CNT1 sample (see Table 1), a Pyrex flask containing the reagent mixture, which consisted of camphor (96% purity in weight, Sigma–Aldrich, Athens, Greece) as carbon precursor and ferrocene (98% purity in weight, Sigma–Aldrich, Athens, Greece) as catalyst, in a 20/1 mass ratio, was connected to the tube nearby the nitrogen inlet. Nitrogen gas flow was used to carry the gas mixture of precursors toward the center of the furnace, where pyrolysis took place at 850 °C, and CNT were grown. For the preparation of CNT2 and CNT3 samples, the catalyst particles were placed on a ceramic boat inside the quartz tube and in the middle of the isothermal zone of the reactor. Firstly, a constant nitrogen flow rate was passed through the quartz tube to remove the air from the system, and, then, the reactor was heated at 700 °C under nitrogen flow. Subsequently, nitrogen was replaced by a mixture of acetylene/nitrogen.

**Table 1:** Sample specifications and characteristics.

Code	Supplier	Production method	Functionalization	Diameter (nm)	Length (μm)	Purity (%)	SSA (m^2^/g)
CNT1	Our lab	CVD	–	60–100	10–30	88–90	37
		Carbon source: camphor					
		Catalyst: ferrocene					

O-CNT1^a^	Our lab	CVD	−COOH	60–100	10–30	>90	–
		Carbon source: camphor					
		Catalyst: ferrocene					

CNT2	Our lab	CVD	–	40–60	10–30	92–94	58
		Carbon source: acetylene					
		Catalyst: Fe particles					

O-CNT2^a^	Our lab	CVD	−COOH	40–60	10–30	>95	–
		Carbon source: acetylene					
		Catalyst: Fe particles					

CNT3	Our lab	CVD	–	20–40	10–30	92–94	96
		Carbon source: acetylene					
		Catalyst: Fe particles					

O-CNT3^a^	Our lab	CVD	−COOH	20–40	10–30	>95	–
		Carbon source: acetylene					
		Catalyst: Fe particles					

REF	Nanothinx (NTX-1)	CVD	–	15–35	10–30	97	104

O-REF^a^	Nanothinx (NTX-5)	CVD	−COOH	15–35	10–30	97	119

A-REF^b^	Nanothinx (NTX-5)	CVD	−CONHCH_2_CH_2_NH_2_	15–35	10–30	97	105

NM-401^c^	JRC	CVD	–	40–90	2–6	75–89	140

a O-CNT 1,2,3 and O-REF indicate the oxidized forms (COOH functionalized) of the corresponding CNT preparations.

b A-REF was amino-functionalized in our laboratory starting from O-REF.

c From data reported in Ref. [54].

**Fig. 1:**
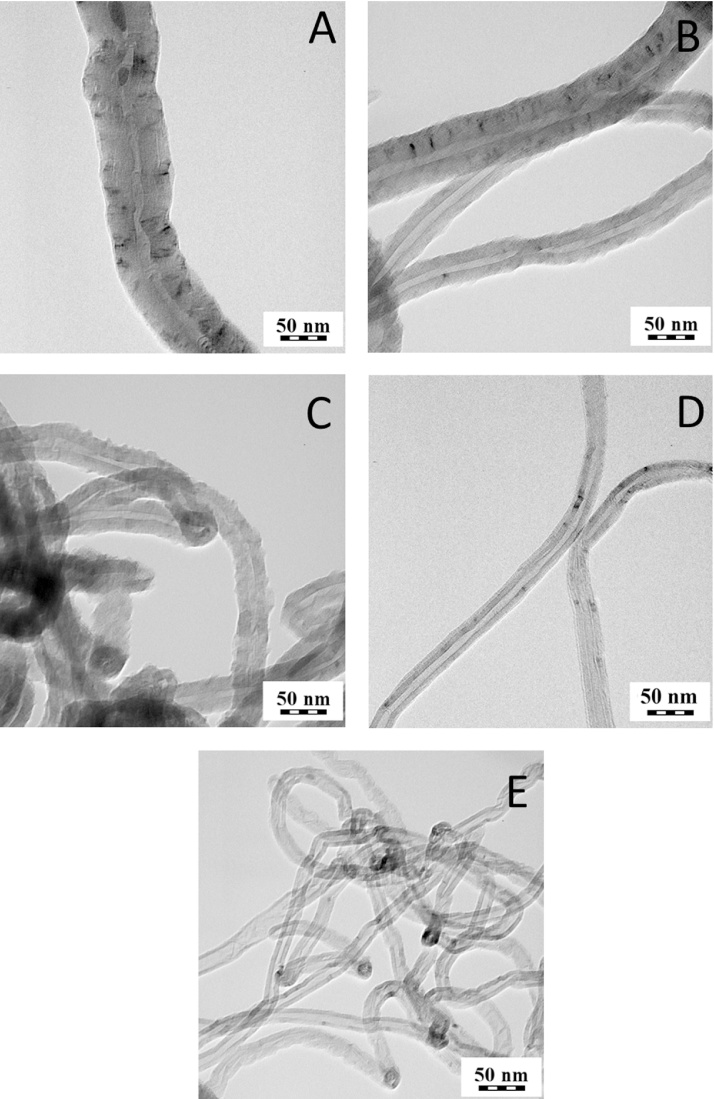
TEM images of CNT1 (a), CNT2 (b), O-CNT2 (c), CNT3 (d), REF(e).

Commercial MWCNT were obtained from Nanothinx S.A. (Patra, Greece). In particular, two types of commercial nanotubes were used in this study: NTX1 (pure MWCNT, REF) and NTX5 (MWCNT functionalized with −COOH groups, O-REF). The physical characteristics of these nanomaterials (according to the technical datasheet) are presented in Table 1. Additionally, a TEM image of the NTX1 nanomaterial is presented in Fig. 1e.

The NM-401 MWCNT preparation was obtained from the JRC Nanomaterials Repository hosting representative industrial nanomaterials (Ispra, Varese, Italy). This nanomaterial is classified as a representative test material (RTM) and includes a (random) sample from one industrial production batch sub-sampled into vials under reproducible (GLP) conditions, with the stability of the sub-samples monitored. A detailed physico-chemical characterization of this material is provided in the specific JRC Report [54].

### 2.2 Carbon nanotubes purification and functionalization process

After the synthesis, the raw products were milled and exposed at atmospheric air flow at 400 °C for 1 h, aiming at the removal of amorphous carbon. Afterwards, they were purified with constant boiling of 5 M HCl in a Soxhlet extractor in order to remove the remaining metal particles. Finally, the purified CNTs were washed with distilled water and dried in oven. To activate the CNT surface with −COOH groups (O-CNT1, O-CNT2 and O-CNT3), an acid solution mixture of 6 M HNO_3_:H_2_SO_4_ in a ratio of 1:3 was used. Then, the CNT/acid mixture (0.15 g CNTs/10 mL acid solution) was stirred for 48 h at 80 °C. The suspension was filtered, and the black powder was washed with ethanol, acetone and distilled water and, eventually, dried in oven.

For the preparation of −CONHCH_2_CH_2_NH_2_ functionalized (amino) MWCNT (A-REF), MWCNT-COOH (O-REF) were stirred in a 20:1 mixture of thionyl chloride (SOCl_2_) and dimethylformamide (DMF) at 70 °C for 1 day. After the acyl-chlorination, MWCNT were centrifuged and washed with anhydrous tetrahydrofuran (THF) for five times. The remaining solid was dried under vacuum. The produced acyl-chlorinated MWCNT reacted with ethylenediamine solution at 100 °C for 2 days. After cooling at room temperature, the MWCNT were washed with ethanol to remove excess diamine. Finally, the material was dried overnight at room temperature and under vacuum [53].

### 2.3 Characterization techniques

#### 2.3.1 X-ray diffraction

The measurements were performed at room temperature with a Bruker D8 Advance Twin Twin X-ray diffractometer (Bruker Corporation, The Woodlands, TX 77381, USA) equipped with a Cu Kα radiation source (wavelength = 1.5418 Å).

#### 2.3.2 Thermogravimetric analysis

The TGA experiments were conducted in oxidative atmosphere (atmospheric air flow: 120 mL/min, heating rate: 5 °C/min) with a Netzsch 409EP instrument (NETZSCH-Gerätebau GmbH, 95100 Selb, Deutschland).

#### 2.3.3 Scanning electron microscopy

The morphology of CNT was determined using a Nova NanoSEM 230 (FEI Inc., Hillsboro, OR, USA) microscope with W (tungsten) filament.

#### 2.3.4 Transmission electron microscopy

TEM measurements were performed with a Tecnai G2 Spirit Twin 12 microscope (FEI Inc., Hillsboro, OR, USA) after the dispersion of CNT in distilled water.

#### 2.3.5 Fourier transform infrared spectroscopy

FT-IR analysis was performed by using a ThermoScientific Nicolet 6700 Fourier Transform Infrared Spectrometer (Thermo Fisher Scientific Inc., Waltham, MA, USA).

#### 2.3.6 Surface area analysis

Brunauer, Emmett and Teller (BET) analysis was performed with a Micromeritics TriStarII 3020 instrument. The specific area was determined by a 5-point BET measurement with UHP nitrogen as the adsorbate and liquid nitrogen as the cryogen.

### 2.4 Suspension Stability Index

A kinetic analysis of suspension stability was performed by monitoring the absorbance of the suspension at 550 nm for different times, using an UV–vis DU650 spectrophotometer (Beckman Coulter SpA, Milano, Italy). Typically, 1 mL of the MWCNT suspension at 128 μg/mL was prepared, and absorbance readings were taken at 5 min intervals for 35 min.

### 2.5 Analysis of adsorbed proteins

To analyze proteins adsorbed to MWCNT, two aliquots of each preparation (REF, O-REF, A-REF), suspended at 1.28 mg/ml in PBS + 0.05% BSA, were diluted 1:10 in DMEM + 10% FBS or in plain DMEM. After an 1 h-incubation at 37 °C under continuous stirring, samples were rapidly centrifuged at 13,000 × *g* and the pellets were washed three times in bidistilled water. The pellets were then suspended in sample buffer (31.25 mM Tris–HCl, pH 6.8, 3% SDS, 10% glycerol 100 mM, DTT, 0.02% bromophenol blue) and boiled for 10 min. Aliquots were used for SDS-PAGE. After the electrophoresis, gels were washed in water and stained with silver staining (Cosmo Bio Co., Ltd., Tokyo, Japan, Cat. No. 423413) according to manufacturer’s instructions.

In parallel, the pellets of other two aliquots, also incubated in DMEM + 10% FBS or in plain DMEM, were suspended in water, and proteins quantified with a modified micro-Lowry procedure [5].

### 2.6 Cells and experimental treatments

Mouse peritoneal monocyte–macrophage cells (Raw264.7 line) were cultured in Dulbecco’s modified Eagle’s medium (DMEM) supplemented with 10% Foetal Bovine Serum (FBS), 4 mM glutamine, and antibiotics (streptomycin 100 μg/mL penicillin, 100 U/mL). Human alveolar carcinoma epithelial cells (A549) were cultured in DMEM supplemented with 10% FBS, 2 mM glutamine, and antibiotics. Calu-3 lung adenocarcinoma cells [23] were cultured in EMEM supplemented with 10% FBS, 2 mM glutamine, 1 mM sodium pyruvate, and antibiotics. The three cell lines were purchased from the Cell bank of the Istituto Zooprofilattico Sperimentale della Lombardia e dell’Emilia (Brescia, Italy). Murine alveolar macrophages (MH-S), a gift of Prof. Dario Ghigo, University of Torino (Italy), were also originally provided by the Cell Bank of the Istituto Zooprofilattico Sperimentale della Lombardia ed Emilia-Romagna (Brescia, Italy) and cultured in RPMI1640 medium supplemented with 10% FBS, antibiotics, l-glutamine (2 mM) and β-mercaptoethanol (0.05 mM). Before and during treatments, all cultures were maintained in a humidified atmosphere of 5% CO_2_ in air in 10-cm dishes.

For treatments, cells were seeded in 96-well dishes at a density of 1.5 × 104 cells/well for viability experiments and in 24-well dishes at a density of 1 × 105 cells/well for nitrite determination. MWCNT were added from stock solutions (2.56 mg/mL) prepared according to a modified Nanogenotox protocol (http://www.nanogenotox.eu/files/PDF/web%20nanogenotox%20dispersion%20protocol.pdf) in which Ca- and Mg-free PBS replaced water + Bovine Serum Albumin (BSA, 0.05%) in order to maintain the physiological osmolality. Doses of materials were expressed in μg/cm^2^ of monolayer; a fixed proportion was maintained between the culture surface and the volume of incubation, so as to maintain the relationship between doses in μg/cm^2^ and doses in μg/mL (dose in μg/cm^2^ × 1.6 = doses in μg/mL) fixed.

No surfactant agent was employed, as we decided to allow aggregation in order to characterize CNT toxicological properties in real-life resembling conditions, *i.e.*, occupational environment.

### 2.7 Cell viability

To evaluate the effects of the different preparations on cell viability, Raw264.7 and A549 cells – two lines representative of macrophages and airway epithelial cells – were treated with the MWCNT to be tested or with the benchmark NM-401 at the indicated doses, using for each preparation a separate dish in which it was tested in parallel with NM-401. Cell viability was measured after 24 h, 48 h, or 72 h of exposure to MWCNT using the resazurin assay [45]. Since it is known that nanomaterials could interfere with cytotoxicity tests, we incubated the dye with each MWCNT preparation at the maximal dose used in the viability experiments (80 μg/cm^2^) in the absence of cells, so as to evaluate autofluorescence; moreover, MWCNT were also added just before the reading, so as to assess possible fluorescence quenching. No interference was detected in either case (data not shown).

### 2.8 Medium nitrite concentration

Nitrite concentration, as a proxy for NO output, was determined in Raw264.7 and MH-S macrophages, which are known to be competent for NO production, through a fluorimetric approach, based on the production of the fluorescent molecule 1*H*-naphthotriazole from 2,3-diaminonaphthalene (DAN) in an acid environment [43]. After 48 h and 72 h of incubation with the materials, 100 μL of medium were transferred to black 96-well plates with a clear bottom (Corning, Cambridge, MA). DAN (20 μL of a solution of 0.025 mg/mL in 0.31 M HCl) was then added and, after 10 min at RT, the reaction was stopped with 20 μL of 0.7 M NaOH. Standards were performed in the same medium from a solution of 1 mM sodium nitrite. Fluorescence (*λ*_ex_ 360 nm; *λ*_em_ 430 nm) was determined with a multimode plate reader Perkin Elmer Enspire (Waltham, Massachussets, USA).

### 2.9 Real time PCR

Total RNA was isolated from Raw264.7 cells with GenElute Mammalian Total RNA Miniprep Kit (Sigma–Aldrich, Milan, Italy). After reverse transcription, aliquots of cDNA from each sample were amplified in a total volume of 25 μL with the Go Taq PCR Master Mix (Promega Italia, Milan, Italy), along with the forward and reverse primers (5 pmol each) reported in Table 2 to evaluate the expression of different pro-inflammatory genes: inducible nitric oxide synthetase (*Nos2*), prostaglandin-endoperoxide synthase 2 (*Ptgs2*), interleukin 6 *(Il6)* and interleukin 1β (*Il1b*)*.* Real-time PCR was performed in a 36-well RotorGeneTM3000, version 5.0.60 (Corbett Research, Mortlake, Australia). For all the messengers to be quantified, each cycle consisted of a denaturation step at 95 °C for 20 s, followed by separate annealing (30 s) and extension (30 s) steps at a temperature characteristic for each pair of primers (see Table 2). Fluorescence was monitored at the end of each extension step. Melting curve analysis was added at the end of each amplification cycle. The analysis of the data was made according to the relative standard curve method [7]. Expression data were reported as the ratio between each investigated mRNA and the Gapdh mRNA.

**Table 2:** Primers used for gene expression studies.

Gene	Forward	Reverse	*T* (°C)
Inducible nitric oxide synthetase (Nos2)	5′-GTT CTC AGC CCA ACA ATA CAA GA-3′	5′-GTG GAC GGG TCG ATG TCA C-3′	57

Prostaglandin-endoperoxide synthase 2 (*Ptgs2*)	5′-GCTCAGCCAGGC- AGCAAATC-3′	5′-ATCCAGTCCGG- GTACAGTCA-3′	56

Interleukin 6 (*Il6*)	5′-TAG TCC TTC CTA CCC CAA TTT CC-3′	5′-TTG GTC CTT AGC CAC TCC TTC-3′	56

Interleukin 1β (*Il1b*)	5′-GCA ACT GTT CCT GAA CTC AAC T-3′	5′-ATC TTT TGG GGT CCG TCA ACT-3′	58

Glyceraldehyde-3- phosphate dehydrogenase (*Gapdh*)	5′-TGT TCC TAC CCC CAA TGT GT-3′	5′-GGT CCT CAG TGT AGC CCA AG-3′	57

### 2.10 Confocal microscopy

The interaction between MWCNT and macrophages and, in particular, the internalization of the materials, was studied in confocal microscopy. Confocal analysis was carried out with a LSM510 Meta scan head integrated with an inverted microscope (Carl Zeiss, Jena, Germany). Samples were observed through a 40× (1.3 NA) oil objective. Image acquisition was carried out in multitrack mode, *i.e.*, through consecutive and independent optical pathways. Vertical sections were obtained with the function Display—Cut (Expert Mode) of the LSM510 confocal microscope software (Microscopy Systems, Hartford, CT).

Reconstructions were performed from *z*-stacks of digital images (minimum 32 confocal sections, *z*-axis acquisition interval of 0.39 mm), processed with the Axiovision module inside 4D release 4.5 (Carl Zeiss, Jena, Germany), applying the shadow or the transparency algorithm.

For microscopy, phagocytosis-competent Raw264.7 macrophages were seeded on four-chamber slides at a density of 15 × 10^4^ cells/cm^2^. After 24 h, monolayers were exposed to MWCNT and incubation prolonged for further 24 h. In the last 20 min of exposure, cells were transferred to serum-free medium supplemented with CellTracker™ Red CMPTX (8 μM, Molecular Probes, Invitrogen) to label the cytoplasm; in the last 5 min 1,5-bis[2-(di-methylamino)ethyl]amino-4,8-dihydroxyanthracene-9,10-dione (DRAQ5^®^, 20 μM, Alexis Biochemicals, San Diego, CA, USA) was also added to the incubation medium to counterstain the nuclei. After the experimental treatments, cell monolayers were rinsed in PBS and fixed with 3.7% paraformaldehyde (PFA) at room temperature for 15 min. After fixation, specimens were mounted on glass slides with fluorescence mounting medium (Dako Italia SpA).

Excitation at 488 nm and reflectance were used to visualize MWCNT (shown in white); excitation at 543 nm and emission recorded through a 580–630 nm band pass barrier filter were used to visualize the cytoplasm (shown in red); excitation at 633 nm and emission through a 670 nm long pass filter were recorded to visualize the nucleus (shown in blue).

### 2.11 Colony forming efficiency (CFE) assay

The CFE test is a clonogenic assay that measures the ability of a single cell to form a colony [25] and can be used to determine cytotoxicity induced by nanomaterials in any adherent cell models. Indeed, since it is a label-free test (non-colorimetric, non-fluorescent), CFE assay lowers the possibility of the occurrence of interference due to nanomaterials [50]. For the test, 10^2^ A549 cells, were seeded in tissue culture dishes (BD Falcon 60 × 15 mm Style, BD Falcon, USA). After 24 h, culture medium was replaced with complete medium supplemented with MWCNT (2.5–5 μg/cm^2^), and the exposure prolonged for 72 h. After medium renewal, incubation was prolonged for further 72 h. Cells were then fixed with a solution of 4% paraformaldehyde for 20 min, washed three times with PBS without Ca and Mg, and stained with a 0.1% crystal violet solution in bi-distilled water. After staining, colonies were counted.

### 2.12 Trans-Epithelial Electrical Resistance

Decrease in the Trans-Epithelial Electrical Resistance (TEER) indicates a perturbation of the barrier function of tight epithelial monolayers, and is a parameter used to evaluate epithelial damage by MWCNT [56], [55], [4], [57] or other nanomaterials [22]. TEER was measured using an epithelial voltmeter (EVOM, World Precision Instruments Inc., Sarasota, FL, USA). For the experiments, Calu-3 cells, an airway epithelial cell line which forms a tight monolayer when cultured in a double chamber culture system, were seeded into cell culture inserts with membrane filters (pore size 0.4 μm) for Falcon 24-well-multitrays (Cat. N° 3095, Becton, Dickinson & Company, Franklin Lakes, NJ, USA), at a density of 2.3 × 10^4^ cells/cm^2^, and grown for 12d until a tight monolayer was formed (TEER > 1000 Ω × cm^2^). MWCNT were added in the apical chamber from a 1 mg/mL stock solution without changing the medium and TEER measured at the indicated times of treatment. TEER changes were expressed as the percentage of the initial value adjusted for control cell monolayers according to Eq. (1) [59]:

(1) $$\text{TEER(\%)=}\frac{{\text{Final TEER}}_{treated}}{{\text{Final TEER}}_{control}}\times \frac{{\text{Initial TEER}}_{control}}{{\text{Initial TEER}}_{treated}}$$

### 2.13 Statistics

Data are expressed as means ± standard deviation (mean ± SD). Statistical analyses were performed with one-way ANOVA with Tukey *post hoc* test. GraphPad Prism^®^ software version 6.00 (GraphPad Software Inc., San Diego, CA) was used. Results were considered significant at *p* < 0.05.

### 2.14 Reagents

Whenever not stated otherwise, all the reagents were provided by Sigma–Aldrich, Milan, Italy.

## 3 Results

### 3.1 MWCNT characterization

After the synthesis, purification and functionalization of MWCNT, their structure, chemical composition and purity were investigated. Table 1 summarizes the features of the MWCNT preparations tested. In Fig. 1, TEM images of selected MWCNT samples are shown. Hollow filamentous structures were revealed for all samples and their specific characteristics were found to vary according to the different production method. In particular, the diameter of MWCNT ranges between 15 and 100 nm depending on the experimental conditions adopted for synthesis. In all cases, the length of CNT ranges between 10 and 30 μm. Iron particles could be seen mostly encapsulated within the core of the MWCNT.

**Fig. 2:**
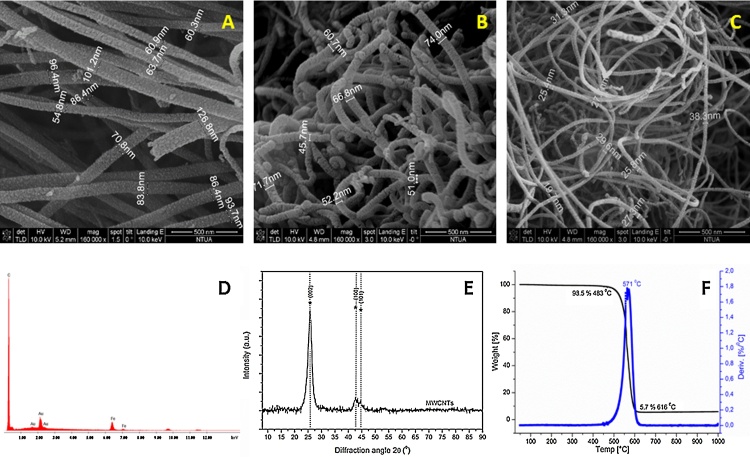
SEM images of (A) CNT1, (B) CNT2 and (C) CNT3; (D) EDS analysis, (E) XRD diagram and (F) TGA graph of CNT3.

SEM images of the CNT preparations produced in our lab, is depicted in Fig. 2, along with a representative EDS analysis, XRD diagram and TGA graph of CNT3 sample. In Fig. 2A–D the SEM images and the corresponding EDS analysis revealed uniform diameter distribution and high purity. The main features of XRD patterns of CNT were close to those of graphite (Fig. 2E); a typical XRD pattern consisted of bands located near the (0 0 2), (1 0 0) and (1 1 0) reflections of graphite. The first peak at 2*θ* ∼ 26° can be attributed to the (0 0 2) reflection of graphite while an asymmetric diffraction peak at 2*θ* ∼ 43° was assigned to the (1 0 0) reflection of graphite, which is typically observed in MWCNT [48], [49]. TGA was used in order to evaluate the thermal stability and the purity degree of the produced CNT (Fig. 2F). The initial weight loss of 1.5%, observed at temperatures up to 480 °C, could be attributed to the burning of amorphous carbon material. The residual weight at the end of the thermal oxidative curve (5.7%) corresponded to the iron catalyst particles. The differential thermogravimetric analysis (DTA) curve showed only one narrow peak at 560 °C indicating the high thermal stability in air atmosphere and the uniform graphitized structure of the CNT produced. The overall purity of CNT3 was around 92.8%. Table 1 includes the TGA data for all the MWCNT preparations. Purity range is that expected for MWCNT prepared with the methods adopted here [6]. There were no substantial differences among the various raw MWCNT preparations for EDX and XRD results (not shown). The specific surface area (SSA) values, obtained with BET (Table 1), indicated that the surface area value increases as the outer diameter of pristine materials decreases [6].

**Fig. 3:**
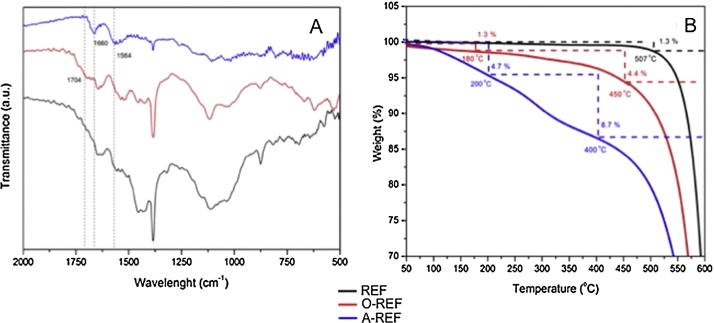
FTIR spectra (A) and TGA graphs (B) of pristine MWCNT (REF), MWCNT–COOH (O-REF) and MWCNT-amino (A-REF) samples.

The functionalization process was studied *via* FT-IR spectroscopy and TG analysis. Specifically, FTIR spectra of pristine MWCNTs (REF), MWCNT–COOH (O-REF) and MWCNT-amino (A-REF) are shown in Fig. 3A. Comparing REF and O-REF with A-REF, some new peaks appeared in the spectrum of A-REF. Especially, the C=O stretching frequencies shifted from 1704 cm^−1^ (MWCNT–COOH) to 1660 cm^−1^ (MWCNT-amino) and a new peak appeared at 1564 cm^−1^, which could be assigned to N—H in plane. These bonds pointed to the existence of a secondary amine on the sidewalls of MWCNT-amino.

Thermogravimetric analysis was applied to quantify the functionalization degree of O-REF and A-REF samples [15]. From Fig. 3B, it is clear that the thermal degradation of the modified CNT is a multistage process. The weight loss occurs at temperatures up to 160–200 °C (∼1.3% for O-REF and ∼4.7% for A-REF) and is associated with the release of moisture which is physically or chemically absorbed. CNT-COOH and CNT-amino samples exhibit a first weight loss at 180–450 °C (∼4.4%) and 200–400 °C (∼8.7%) corresponding to the burning of carboxyl and −CONHCH_2_CH_2_NH_2_ groups, respectively. The second loss (temperatures above 400–450 °C) is attributed to the CNT burning [15], [17], [71]. The approximate molar% functional groups on CNT were estimated [71] equal to ∼1.3 mol% for both cases, indicating the high conversion of the functionalization process.

Overall, the three CNT preparations do not present significant differences in their specific area. BET analysis revealed the following results: 104 m^2^/g (REF), 119 m^2^/g (O-REF) and 105 m^2^/g (A-REF). It can be observed that after −COOH functionalization, MWCNT showed a slight increase in their specific area. This may be attributed to the oxidation treatment which creates sidewall openings and defects. On the other hand, the attachment of amino functional groups does not lead to a comparable increase, since the groups added are more bulky than −COOH ones, blocking CNT pores [6], [8].

The surface charge of the MWCNT preparations can be estimated taking into account the Point of Zero Charge (PZC) theory [3]. According to this theory, pH is considered to be the most important factor which determines the surface charge of CNTs, with the surface charge negative when the solution pH is higher than the PZC value, while otherwise becomes positive. The PZC can be estimated to be 6.6 for the pristine REF, 3.1 for O-REF and 10.8 for A-REF [3], indicating that at the pH of the culture medium (roughly 7.4) REF and O-REF are mainly negative and A-REF mainly positive.

**Fig. 4:**
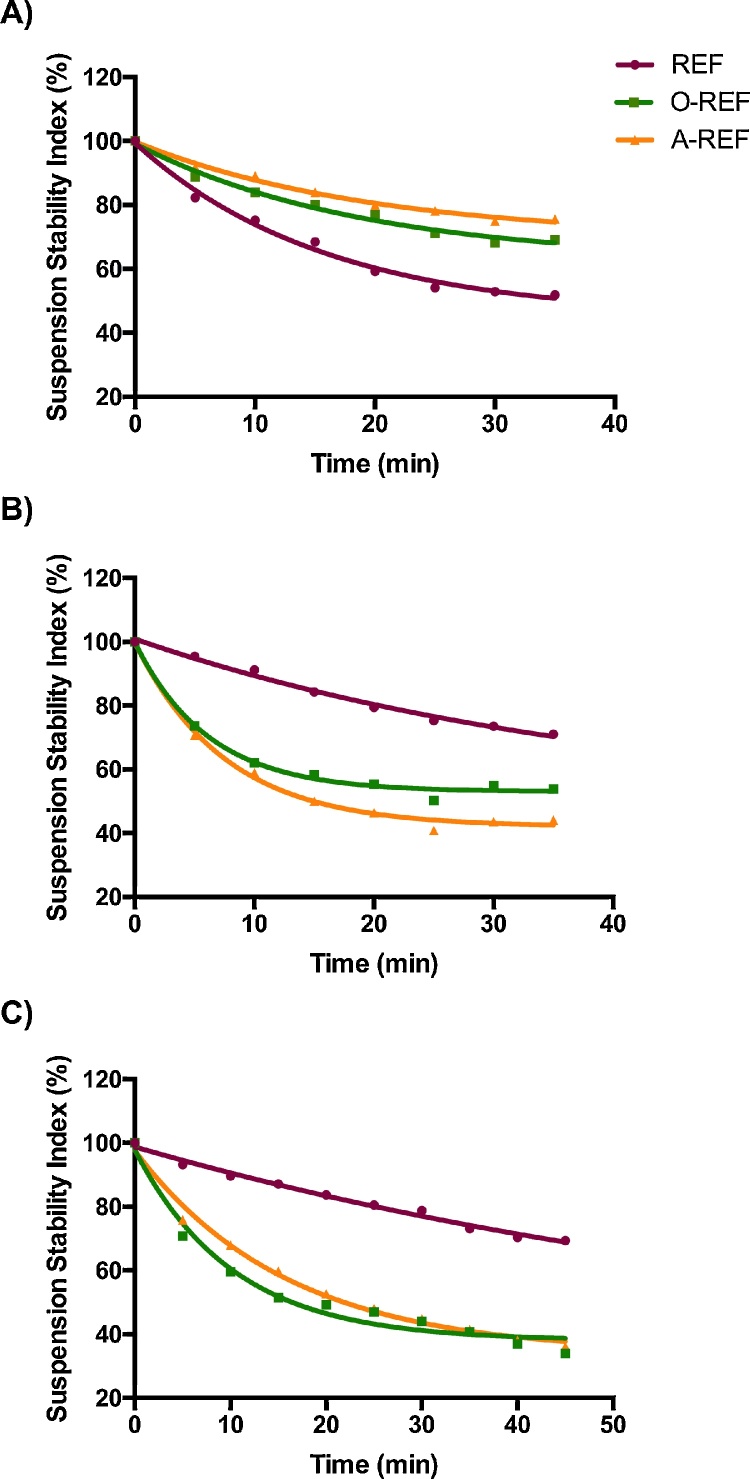
The Suspension Stability Index of the three MWCNT preparations at 128 μg/mL was expressed as the % of the initial absorbance at the indicated times. The absorbance measurements were carried out as described in Section 2.4. (panel A) MWCNT were suspended in PBS (Ca- and Mg-free). (panel B) MWCNT were suspended in a solution of 0.05% BSA in PBS (Ca- and Mg-free). (panel C) MWCNT were suspended in growth medium (phenol-red free DMEM) supplemented with 10% FBS. The figure shows a representative experiment, repeated twice with comparable results.

The Suspension Stability Index was determined for REF, O-REF and A-REF at the maximal concentration used in the biological experiments (128 μg/mL, corresponding to a monolayer exposure of 80 μg/cm^2^) according to the method described by Wang et al. [67]. The results (Fig. 4) indicate that the suspension of pristine MWCNT (REF) was less stable than carboxyl- (O-REF) or amino-functionalized MWCNT dispersed in PBS (panel A). Comparable results have been obtained in 0.9% NaCl solution (data not shown). However, this pattern was reversed upon the addition of proteins, since REF suspension was more stable than O-REF and A-REF suspensions in either PBS + BSA or medium (DMEM) + 10% FBS. These data suggest that larger agglomerates are formed by O-REF and A-REF MWCNT in protein-rich media. The stability of REF and A-REF samples was roughly comparable in the two protein-rich suspension media, while O-REF suspensions were less stable in PBS + BSA than in medium + FBS.

### 3.2 Interaction of MWCNT with proteins

**Fig. 5:**
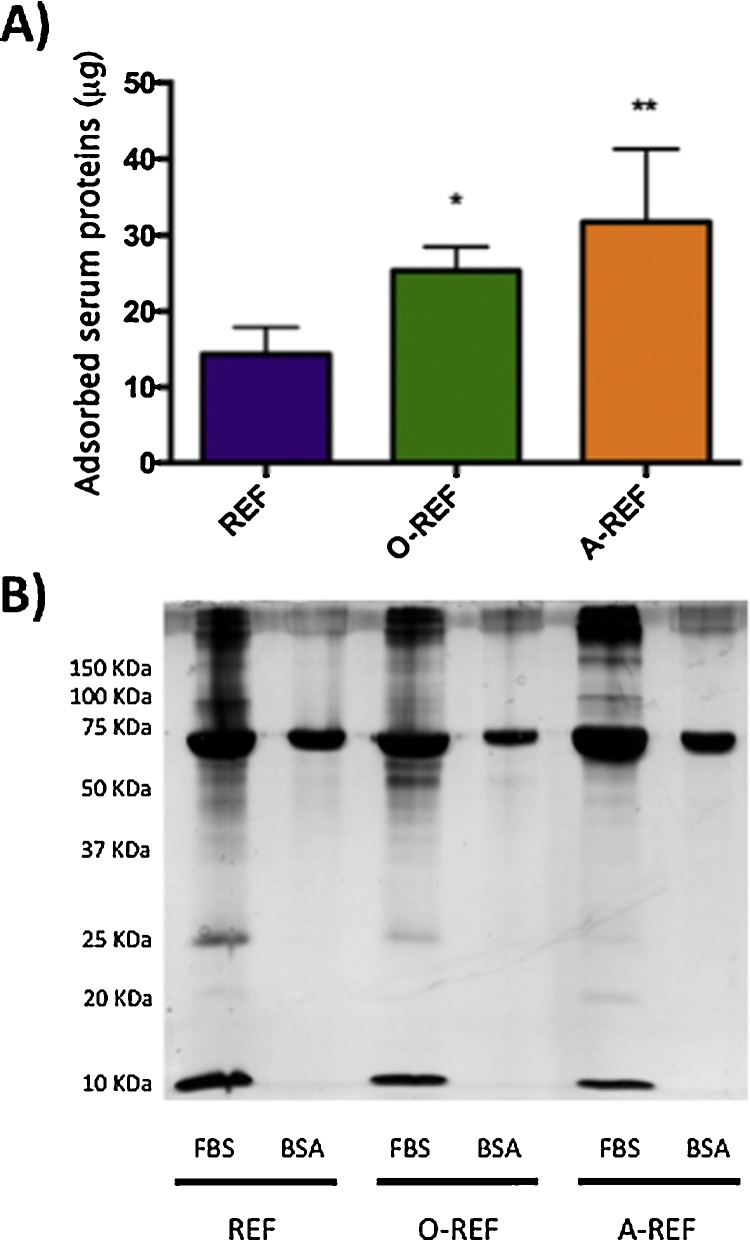
Protein adsorption on MWCNT. MWCNT preparations, dispersed in 0.05 wt% BSA, were incubated at the concentration of 128 μg/mL for 1 h in culture medium with or without 10% FBS. At the end of the incubation, the suspensions were centrifuged, and adsorbed proteins quantified (A) or separated and stained (B) as described in Section 2. For (A), data are means ± SD of 4 independent determinations. *p < 0.05, **p < 0.01 *vs.* REF. For (B) a representative experiment, performed twice with comparable results is shown.

In order to evaluate the adsorption of serum proteins to MWCNT, REF, O-REF and A-REF were incubated with culture medium supplemented with 10% FBS. Proteins adsorbed to MWCNT were then quantified with a colorimetric method or detected with silver staining after polyacrylamide gel electrophoresis (Fig. 5). Quantification of adsorbed serum proteins (panel A) evidenced that, although all the three MWCNT preparations bound serum proteins, the amount of adsorbed proteins was significantly higher for functionalized MWCNT than for pristine counterpart (A-REF > O-REF > REF). The pattern of adsorbed serum proteins (lanes 1, 3, 5) was different for the three MWCNT preparations. In particular, when MWCNT were incubated with serum, A-CNT adsorbed more BSA than the other two preparations but many other bands were fainter. Moreover, while few bands were clearly more abundant in O-REF, several others were less evident in functionalized than in pristine MWCNT.

### 3.3 Cell viability

The effect of the different MWCNT preparations (dose range 2.5–80 μg/cm^2^) on the viability of Raw264.7 macrophages was tested with the resazurin assay every 24 h up to a 72 h-exposure to the materials (Table 3). NM-401 MWCNT, which we have found endowed with marked cytotoxic effects toward Raw264.7 macrophages (unpublished results), were used as a benchmark. Significant effects on cell viability were observed only at 72 h of incubation. However, while NM-401 produced a clear cut dose-dependent decrease of cell viability, only moderate effects were observed for the other MWCNT. In particular, no decrease in viability was observed with MWCNT wider than 40 nm. On the other hand, MWCNT preparations with diameters between 15 and 40 nm produced a decrease in viability, although no material, but the benchmark NM-401, caused a viability loss larger than 50%. The direct comparison among the IC_20_ values obtained with pristine MWCNT of different diameter indicated that cytotoxicity was roughly inversely proportional to diameter (REF = CNT3 > CNT2 = CNT1). Given that NM-401 are long, needle-like, while our samples are even longer, but entangled, we can speculate that cytotoxic properties are also associated with MWCNT shape, although this issue has not been further investigated.

**Table 3:** Effect of MWCNTs on cell viability of Raw264.7 macrophages.

Code	IC_50_ (μg/cm^2^)	IC_20_ (μg/cm^2^)
CNT1	>80	>80
O-CNT1	>80	>80
CNT2	>80	>80
O-CNT2	>80	>80
CNT3	>80	23.9
O-CNT3	>80	46.9
REF	>80	29.1
O-REF	>80	39.2
A-REF	ND	ND
NM-401	26.4	9.9

ND, not determined.

Functionalization with -COOH groups did not change appreciably MWCNT effects on cell viability for CNT1 and CNT2, while IC_20_ values of the functionalized O-CNT3 and O-REF were larger than those obtained for the corresponding pristine preparations CNT3 and REF, indicating a mitigating effect.

**Fig. 6:**
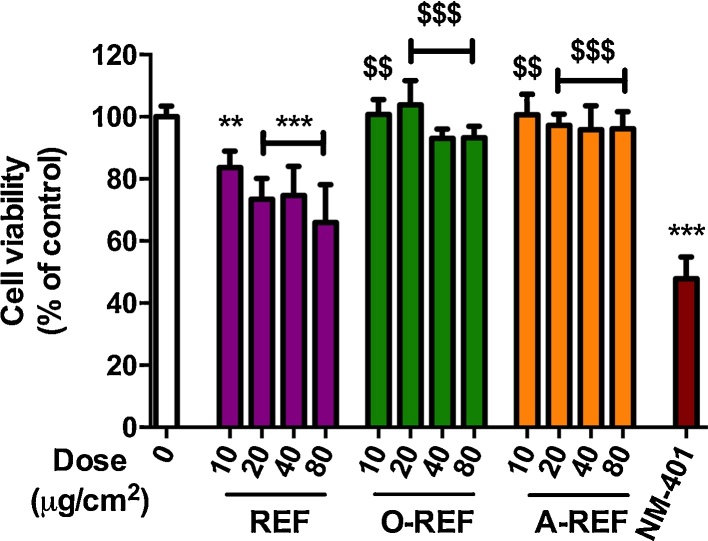
Cell viability of A549 cells exposed to MWCNT. Cells were treated with increasing doses of MWCNT (range dose 10–80 μg/cm^2^ for REF, O-REF and A-REF; 80 μg/cm^2^ for NM-401). After 48 h cell viability was determined with the resazurin assay. 0, control untreated cells. Data are means ± SD of 6 indipendent determinations obtained in two separate experiments. **p < 0.01, ***p < 0.001 *vs.* control, untreated cells (0); $$*p* < 0.01, $$$*p* < 0.001 *vs.* equivalent doses of REF.

To ascertain if the mitigating effects of functionalization were specific for macrophages, we extended the study to the human epithelial airway cells A549, a cell model widely adopted in toxicological studies. On these cells, we studied three preparations (pristine, carboxyl-functionalized and amino-functionalized) of the thinnest MWCNT listed in Table 1. The results (Fig. 6) revealed that the pristine REF determined a clear cut dose dependent decrease of cell viability up to 35% at the maximal dose (80 μg/cm^2^). A much slighter, but still significant decrease was instead observed in the case of the COOH-functionalized preparation (up to 7% at the maximal dose). On the contrary, A-REF did not show a significant cytotoxic effect even at the highest dose tested (80 μg/cm^2^). As expected, the benchmark NM-401, used at the dose of 80 μg/cm^2^, determined a marked decrease (around 50%) of cell viability. Thus, also in epithelial cells functionalization with either -COOH or amino groups mitigated the effect on cell viability.

**Fig. 7:**
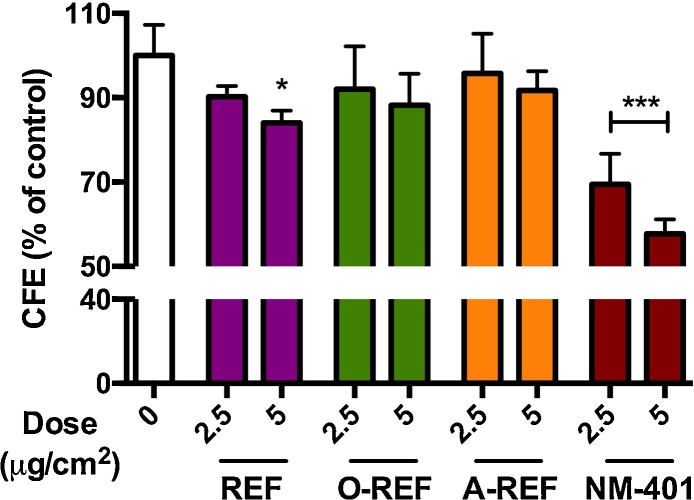
Colony forming efficiency of MWCNT-treated A549 cells. Cells were seeded at a concentration of 100 cells/plate and treated as described in Section 2.11. Data are means of 4 indipendent determinations. *p < 0.05, ***p < 0.001 *vs*. control, untreated cultures.

To deepen the investigation of the effect of functionalization on cell viability, the different MWCNT preparations (2.5–5 μg/cm^2^) were studied with a CFE assay on A549 (Fig. 7). Only the REF preparation determined a significant decrease of colony number at the highest dose tested of 5 μg/cm^2^, while the other MWCNT did not produce significant effects. The rank of the effect was confirmed at 10 μg/cm^2^ (REF 78.3 ± 6.4, *n* = 3, *p* < 0.05; O-REF 87.2 ± 10.3, *n* = 3, n.s.; A-REF 94.7 ± 15.9, *n* = 3, n.s.). The benchmark material NM-401 induced a significant dose dependent decrease of colony number up to 45% at the highest dose tested of 5 μg/cm^2^.

### 3.4 Macrophage activation

Macrophages play a pivotal role in determining the final outcome of the interaction between the organism and the nanomaterials. In particular, if the exposure to the nanomaterial leads to macrophage activation, this triggers an inflammatory response.

**Fig. 8:**
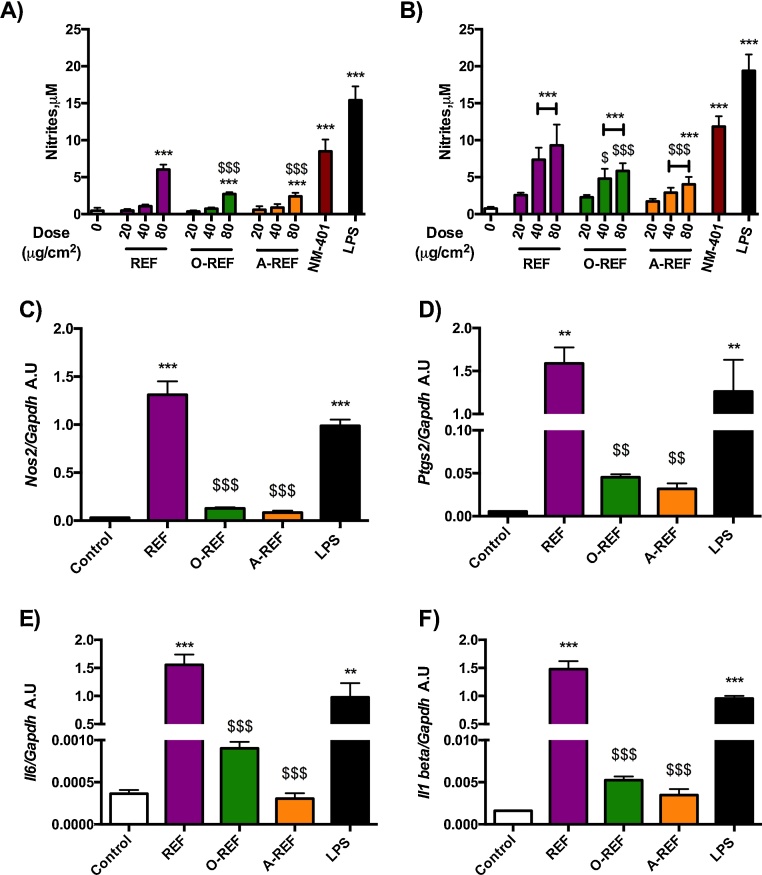
Expression of pro-inflammatory markers in Raw264.7 exposed to MWCNT. (panels A and B) Nitrite concentration in cell culture medium of Raw264.7 cells exposed to MWCNT. Raw264.7 cells were treated with MWCNT (REF, O-REF and A-REF: range dose 20–80 μg/cm^2^, NM-401: 80 μg/cm^2^). After 48 h (panel A) or 72 h (panel B), the nitrite concentration was determined in cell culture medium. Lipopolysaccharide (LPS, 10 ng/ml) was used as a positive control. Data are means ± SD of 8 independent determinations obtained in two experiments. (panels C, D and E) Expression of *Nos2* (panel C), *Ptgs2* (panel D), *Il6* (panel E), and *Il1b* (panel F) in cells treated with MWCNT. Raw264.7 cells were treated with the indicated materials at the dose of 80 μg/cm^2^. After 18 h mRNA was extracted and gene expression was analyzed with RT-PCR. Data are means of 2 independent determinations. **, ***p < 0.01, *p* < 0.001 *vs.* control, untreated cells. $, $$, $$$*p *< 0.05, *p* < 0.01, *p *< 0.001 *vs.* REF at the same dose.

Fig. 8 reports data on the production of NO, a major inflammatory mediator produced by activated macrophages, and on the expression of pro-inflammatory genes in Raw264.7 cells exposed to MWCNT. Panels A and B report data on NO production, assessed from the nitrite concentration in the medium after 48 h (A) or 72 h (B) of treatment of Raw264.7 cells with increasing doses of REF, O-REF and A-REF (range 20–80 μg/cm^2^). The MWCNT tested induced dose- and time-dependent increases in NO production with different potencies. In particular, the pristine REF determined the strongest macrophage activation with a rank of pro-inflammatory activity REF > O-REF ≅ A-REF. This rank was observed for the stimulation of NO production also in a different model, the alveolar murine macrophages MH-S, which have a lower basal NO output than Raw264.7 cells but exhibit qualitatively comparable changes. In these cells, nitrite medium concentration in untreated cells was 0.18 μM ± 0.16 and increased by 12-fold (*p* < 0.001) with REF, 3-fold (not statistically significant) with O-REF, and 2-fold (not statistically significant) for A-REF. The same rank was also maintained for the induction of the *Nos2* gene in Raw264.7, which encodes for the inducible form of nitric oxide synthase (Nos2) (panel C). The higher effect of REF on macrophage activation was not limited to NO production and *Nos2* induction but involved also the expression of three other important pro-inflammatory genes: *Ptgs2* (panel D), which encodes for the inducible form of cyclooxygenase; *Il6*, the gene for the inflammatory cytokine interleukin-6 (Panel E); and *Il1b* for IL-1β (panel F).

**Fig. 9:**
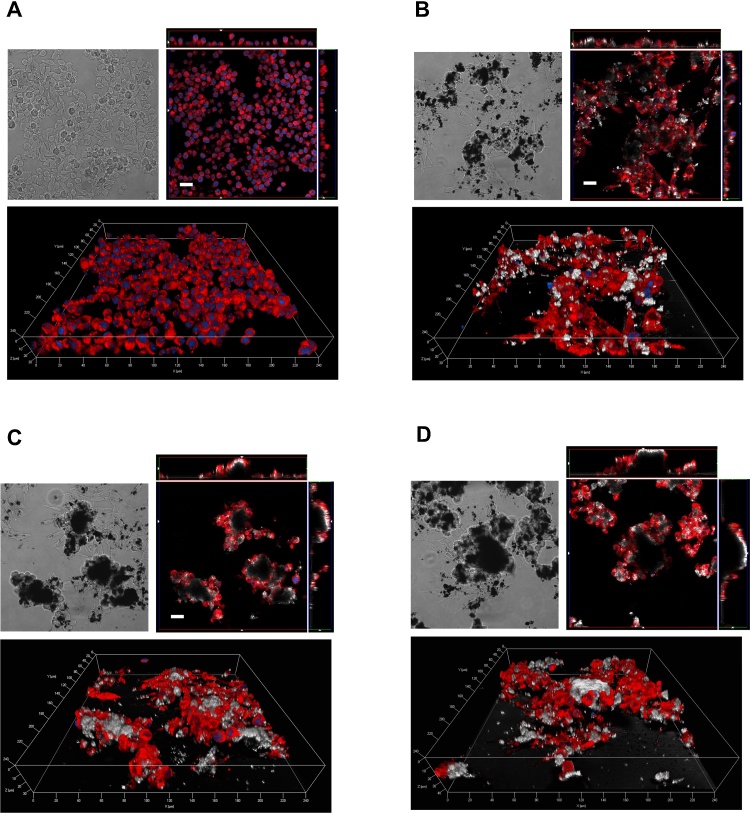
Contrast phase (top left) and confocal images (top right and bottom) of Raw264.7 cells exposed to REF (panel B), O-REF (panel C) and A-REF (panel D) MWCNT. Cells were treated with 80 μg/cm^2^ of each MWCNT preparation for 24 h and labelled as described in Methods. The same representative fields are shown in contrast phase (left) and confocal microscopy (right). For each condition, a single horizontal confocal section is shown along with two orthogonal projections. White, MWCNT (reflection mode); blue, nuclei; red, cytoplasm. Bars = 20 μm. (For interpretation of the references to color in this figure legend, the reader is referred to the web version of this article.)

Fig. 9 reports phase contrast and confocal images of Raw264.7 cells treated with 80 μg/cm^2^ of MWCNT. In the case of REF (panel B), MWCNT appear to be more dispersed with smaller agglomerates than those present in the case of O-REF (panel C) and A-REF (panel D). In all cases, macrophages appear to adhere to the MWCNT agglomerates, to climb on the material, as indicated by the orthogonal projections, and to become markedly elongated, a morphological hallmark of activation. The smaller size of REF agglomerates facilitates macrophage phagocytic activity, as demonstrated by the more diffuse presence of intracellular MWCNT in this preparation. On the contrary, large agglomerates are not internalized by macrophages.

### 3.5 Epithelial barrier competence

**Fig. 10:**
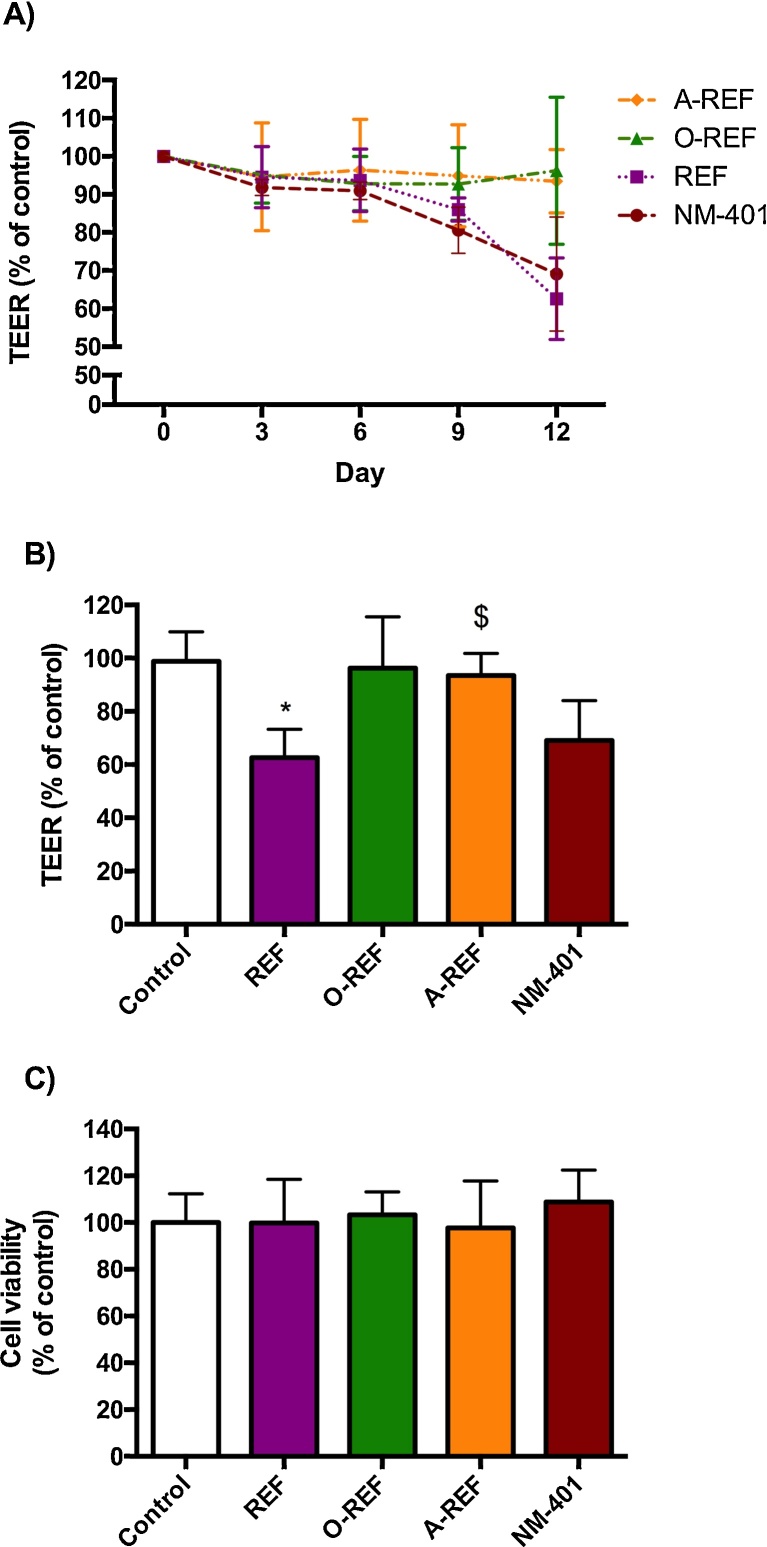
TEER and cell viability of Calu-3 cell monolayers. Calu-3 cells were seeded on permeable filters as described in Section 2.12. After a tight monolayer had formed, MWCNT (80 μg/cm^2^) were added to the apical compartment, and exposure prolonged for up to 12 days. At the indicated times, TEER was determined, as described under Methods. (A) TEER (% of control) recorded every 3d up to 12d. (B) TEER (% of control) at day 12. (C) Cell viability was assessed in the same wells used for TEER determinations with the resazurin assay. For all panels, data are means ± S.D of 4 independent determinations. *p < 0.05 *vs.* control, untreated cultures. The experiment was performed twice with comparable results.

Fig. 10 (panel A) reports the time course of changes in the Trans-Epithelial Electrical Resistance (TEER) of Calu-3 cell monolayers exposed to MWCNT (80 μg/cm^2^) up to 12d. REF significantly lowered TEER at 12d with a decrease of 20% compared to control monolayers. Conversely, O-REF and A-REF did not produce any significant effect, while the benchmark material NM-401 produced the largest decrease in TEER (>30% at 12d of treatment). Cell viability, monitored with resazurin assay in the same wells where TEER evaluation was performed, exhibited no significant changes under any experimental condition (panel C).

## 4 Discussion

Although relatively less studied than the toxicological properties of SWCNTs, the biological effects of MWCNT have been also repeatedly investigated *in vitro* and *in vivo*. According to the literature, various factors influence the biological effects of MWCNT and have the potential to modulate their biological effects [28]. In particular, a correlation has been reported between MWCNT toxicity, their size [28], [34], contamination with metal catalysts [1], and surface functionalization [28].

However, it is difficult to find examples in literature where differences between two MWCNT preparations are limited to one parameter, allowing a clear cut assessment of its contribution to the toxicological properties of the nanomaterial. In this study, we have preliminarily compared a panel of MWCNT of comparable length (> 10 μm), which differ for diameter (range 15–100 nm). Each preparation was available in pristine form or after COOH-functionalization. Low acute cytotoxicity was observed with both types of materials in macrophages, although an increased effect was detected for thinner MWCNT. In general, while it is usually accepted that the longer are the MWCNT tested, the higher is their toxicity [28], [34], [61], contrasting reports exist on the role of diameter. According to some authors, the larger is MWCNT diameter, the higher is the toxicity reported on cells or with *in vivo* models [28], whereas the converse has been also described [21].

Using one of the MWCNT preparations endowed with significant effects on cell viability, we found that the presence of either carboxyl or amino groups mitigated the effects. The extension of the study to other endpoints indicated that the mitigating effect was not limited to decrease in cell viability of MWCNT. In particular, surface functionalization decreased the activating effects of MWCNT on macrophages, an endpoint related to the pro-inflammatory activity of MWCNT *in vivo.* This result is at variance with a recently published report [70] in which, however, the preparation of functionalized MWCNT used consisted in nanotubes much shorter than the pristine ones. Also the perturbation of the epithelial barrier, assessed through TEER measurements in airway epithelial cell monolayers, and the decrease in clonogenic activity were clearly more evident for pristine MWCNT than for functionalized derivatives.

For these endpoints, mitigation of toxic effects was observed with either carboxyl- or amino-functionalized MWCNT. As far as we know, while carboxyl-MWCNT have been repeatedly tested, the effects of amino-functionalization on MWCNT toxicity have never been investigated in depth. However, it is interesting that mitigation occurs independently of the net charge present on the MWCNT surface. However, we noted that the three materials more closely compared in this study, REF, O-REF and A-REF, also differed for their agglomeration tendency, with REF forming relatively small agglomerates, while large MWCNT agglomerates were evident in cell cultures treated with carboxyl or amino-functionalized counterparts (Fig. 8). Different tendency to agglomeration of the three preparations tested is consistent with the different sedimentation rates exhibited in both albumin supplemented saline solution and in complete, serum-supplemented culture medium (Fig. 4). These data are similar to the results of Hamilton et al. [28] who, comparing the effects of three preparations of pristine and COOH-functionalized MWCNT, found that functionalized MWCNT formed larger agglomerates than pristine counterparts when suspended in cell culture medium. Interestingly, the effect was particularly evident for the MWCNT of a length comparable to that of the materials tested here. The presence of larger agglomerates has the obvious consequence of lowering the surface available for the interaction between cells and MWCNT. Thus, if it were expressed per surface area, it would be possible that the actual dose was actually lower for O-REF and A-REF than for REF. Moreover, small agglomerates of REF, but not the larger ones of O-REF and A-REF, could be internalized more efficiently than larger (Fig. 9), a difference of obvious importance in explaining the smaller pro-inflammatory activity of functionalized MWCNT.

The reason why functionalized MWCNT exhibited higher agglomeration tendency has not been specifically investigated here. However, from the data reported in Fig. 4, it is evident that the sedimentation of the MWCNT is profoundly influenced by the presence of proteins in the suspension medium, suggesting a role for the protein corona. Interestingly, recent data also indicate that COOH-functionalization has marked effects on the type of proteins adsorbed by MWCNT [60]. It is, therefore, likely that the greater agglomeration tendency exhibited by functionalized MWCNT in protein-rich media depends on their different interaction with proteins.

The role of agglomeration in MWCNT toxicity is still incompletely defined. *In vitro* experiments have indicated that agglomerated MWCNT are usually poorly toxic [69]. Greater toxicity of dispersed compared to agglomerated MWCNT has been confirmed with *in vivo* models [66], although it should be stressed out that the experimental condition adopted to improve dispersion in that paper, *i.e.* grinding, also substantially modifies the size of the materials.

Most of the contributions that investigate toxicity mitigation by MWCNT functionalization do not address the possible influence of surface modification on agglomeration tendency. A notable exception is represented by the recent contribution by Ursini et al. [65]. These authors have investigated the effects of pristine MWCNT, MWCNT-OH and MWCNT-COOH on two epithelial models (BEAS-2B and A549), showing different entry mechanisms and differential toxicity of the three preparations. The size of agglomerates, also determined in medium, was comparable for pristine and MWCNT-COOH. Therefore, these authors did not further consider agglomeration as a possible toxicity modulator. However, at variance with the materials used here, pristine MWCNT were much longer than MWCNT-COOH, thus rendering difficult a straightforward comparison between the two preparations. Conversely, Hamilton et al. [28], comparing MWCNT preparations of comparable length, measured the size of agglomerates formed by pristine and functionalized MWCNT in culture medium and found that COOH functionalization actually increases the size of agglomerates. However, the possible relationship between this behavior and the reduced bioactivity of functionalized MWCNT, exhibited both *in vitro* [28] and *in vivo* [58], was not been further discussed.

In conclusion, this contribution, while confirming that diameter is inversely related to the bioactivity of MWCNT preparations, indicates that the mitigating effects of carboxyl- or amino-functionalization may be, at least in part, attributable to the greater tendency of functionalized MWCNT to form large agglomerates in protein-rich biological fluids.

## Competing interests

The authors declare no competing financial interests. M.A., M.G.B., D.K.P, M.A.K, A-F.T. equally contributed to this manuscript.

## Transparency document

The http://dx.doi.org/10.1016/j.toxrep.2016.01.011 associated with this article can be found in the online version.

Transparency Document
